# Effect of propolis extract on clinical parameters and salivary level of matrix metalloproteinase 8 in periodontitis patients: A randomized controlled clinical trial

**DOI:** 10.34172/japid.2021.013

**Published:** 2021-09-26

**Authors:** Reyhaneh Eghbali Zarch, Mitra Askari, Hamidreza Boostani, Iraj Mirzaii-Dizgah

**Affiliations:** ^1^Faculty of Dentistry, Tehran University of Medical Sciences, Tehran, Iran; ^2^Oral and Maxillofacial Pathology Department, Faculty of Dentistry, Tehran University of Medical Sciences, International Campus, Tehran, Iran; ^3^Periodontist, Private Practice, Tehran, Iran; ^4^Physiology Department, Aja University of Medical Sciences, Tehran, Iran

**Keywords:** Matrix metalloproteinase-8, periodontitis, propolis, randomized controlled trial, saliva

## Abstract

**Background:**

Periodontitis is the bacterial-induced inflammation of tooth-supporting structures. Local antibacterial agents are used as adjunctive therapy in the treatment of periodontitis. This study aimed to compare the effect of subgingivally delivered propolis extract (a resin produced by honey bees) with chlorhexidine (CHX) mouthwash on clinical parameters and salivary levels of matrix metalloproteinase 8 (MMP-8) in periodontitis patients.

**Methods:**

Twenty-eight periodontitis patients in stage II or III and grade B, who had deep periodontal pockets (≥4 mm) around at least three non-adjacent teeth, were divided into two groups. In the control group, patients were prescribed 0.2% CHX mouthwash twice a day for two weeks. In the 20% propolis hydroalcoholic group, subgingival irrigation was performed twice a week for two weeks. Clinical parameters were measured at baseline and after two months. Salivary samples were collected from the propolis and control groups at baseline and two months later to assess MMP-8 levels using the enzyme-linked immunosorbent assay. Additionally, salivary samples from 12 periodontally healthy subjects were used to determine the normal levels of MMP-8. The data were analyzed using SPSS. P<0.05 was considered the level of significance.

**Results:**

In the healthy group, the mean salivary levels of MMP-8 were significantly lower than that in the control and propolis groups at baseline (P<0.001). The results indicated a significant improvement in clinical parameters (P<0.001) in the propolis group compared to the control group, while MMP-8 levels decreased significantly in both groups (P<0.001).

**Conclusion:**

Propolis is recommended as adjunctive therapy for periodontitis patients. Clinical trials registration code: IRCT2016122030475N3.

## Introduction

Periodontitis is the inflammation of tooth-supporting structures due to subgingival inflammation secondary to bacterial plaque accumulation in the region.^1^ Periodontitis treatment includes elimination or reduction of subgingival microflora through scaling and root planing (SRP).^2^ To reduce the need for periodontal pocket elimination surgery, it is recommended that antimicrobial agents be used in conjunction with mechanical instruments.^3^ For more than three decades, chlorhexidine (CHX) has been used to treat periodontal diseases and remains the gold standard anti-plaque mouthwash. It is safe and non-toxic and has strong local antibacterial properties. Nevertheless, its long-term use has some side effects, such as staining of teeth.^4^

Matrix metalloproteinases (MMPs) are a family of protease enzymes with a role in extracellular matrix degradation and reconstruction. In healthy subjects, the activity of MMPs is regulated by inhibitors. Released MMPs are activated when necessary to degrade the extracellular matrix. Periodontopathogens contribute to an imbalance between MMPs and their inhibitors, resulting in periodontal destruction. There is evidence supporting an association between the salivary levels of MMP-8 and periodontal disease clinical parameters. Increased salivary levels of MMP-8 are significantly correlated with periodontitis.^5-7^ Furthermore, specific biomarkers were emphasized in the new classification of periodontal diseases and conditions since they might improve diagnostic accuracy, and their threshold might be incorporated into periodontitis assessment as soon as evidence becomes available.^8^

Propolis is a complex resin compound produced by honey bees. The composition of propolis might vary depending on its botanical origin; however, there is evidence of antibacterial activity in all types of propolis.^9,10^ Propolis has strong inhibitory effects on Porphyromonas gingivalis and other periopathogenic bacterial strains, nine fungi, three protozoa species, and a wide range of viruses.^11,12^ Due to the strong anti-infective activity of propolis, it is referred to as a natural antibiotic. Anti-inflammatory effects of propolis are also effective in improving clinical parameters by modulating cytokines and inflammatory mediators and inhibiting the production of transforming growth factor-β, histamine, and prostaglandins.^13^ Propolis has immunomodulating and topical anesthetic effects and improves wound healing.^14,15^ It can also decrease the prevalence of dental caries^16^ and pulpal inflammation.^17^ Some clinical trials have reported additional improvements in periodontal status through the topical use of propolis.^18,19^ To date, there is no clear consensus on the effect of topical propolis administration as an adjunctive therapy.

To the best of our knowledge, no studies have investigated the impact of propolis on changes in salivary MMP-8 levels. Therefore, this study aimed to compare the effectiveness of propolis extract with CHX as an adjunctive treatment for periodontitis by evaluating their effect on clinical parameters and salivary levels of MMP-8.

## Methods

### Patient selection

This randomized, single-blind controlled clinical trial, following CONSORT guidelines, was conducted on 37 periodontitis patients in stage II or III in terms of severity and grade B in terms of risk of progression, who volunteered to participate in the study and were referred to the Periodontology Department of Tehran University of Medical Sciences, School of Dentistry, International Campus. The study was approved by the Vice-Chancellor for Research, Tehran University of Medical Sciences (Ethical code: IR.TUMS.REC.1394.1066) and registered in the Iranian Registry of Clinical Trials (code: IRCT2016122030475N3). This research was conducted in full accordance with the World Medical Association Declaration of Helsinki and with the ethical standards of the committee in charge of human experimentation (institutional and national). All the participants were briefed about the study and signed written informed consent forms.

### Inclusion criteria

Patients diagnosed with periodontitis stage II or III in terms of severity and grade B in terms of risk of progression, aged 25‒65, who had completed initial periodontal therapy and had deep periodontal pockets (≥4 mm) around at least three non-adjacent teeth. Etiologic factors for biofilm formation (such as caries and defective restorations) were eliminated, and hopeless teeth were extracted.

### Exclusion criteria

The exclusion criteria consisted of periapical changes, systemic conditions requiring antibiotic prophylaxis, or disease affecting the progression or treatment of periodontitis. In addition, other exclusion criteria were consumption of antibiotics, anti-inflammatory drugs, anticonvulsants, immunosuppressive drugs, or calcium channel blockers in the last three months, pregnancy, smoking, and use of mouth rinses during treatment.

### Study design and clinical examination

All the patients received oral hygiene instructions during the initial visit. Ideally, when emergency treatment is not needed, patients should be given at least 1-2 weeks to improve their oral hygiene.^20^ Therefore, two weeks after the primary visit, if the patient’s O’Leary index reached a plaque index of at least <10%,^21^ clinical examinations were recorded by a single experienced periodontist blinded to the patient group allocation. Clinical parameters, including gingival index (GI),^22^ bleeding on probing (BOP), periodontal pocket depth (PPD), and attachment loss (AL),^21^ were measured at baseline (before initiating treatment) and two months later. After that, there were more visits scheduled for patients who required further treatment.

For every 10 patients, 10 envelopes were allocated, five of which were propolis irrigation, and the remaining were CHX mouthwash. Thus, patients were randomized to the test (propolis) and control (CHX) groups. After randomization of the first 10 patients, this procedure was repeated.

### Periodontal therapy

In the control group, 0.2% CHX mouthwash (Shahredaru, Iran) was prescribed twice daily for two weeks. In the propolis group, subgingival irrigation was performed in periodontal pockets with 3 mL of the hydroalcoholic solution of propolis extract twice a week for two weeks.^23^ Therefore, the current study compared two well-established protocols of adjunctive periodontitis treatment with no regard to the dose of agents.

Salivary samples were collected at the onset of treatment and two months later just before recording the clinical parameters.^24^ Patients were told not to eat and drink two hours before collecting saliva. The oral cavity was inspected for debris, and if present, the patients were asked to brush their teeth without toothpaste. Salivary samples were collected from 8 to 11 a.m. Approximately 3 mL of unstimulated saliva was collected from each patient in 5-mL sterile tubes. The samples were frozen and subjected to the enzyme-linked immunosorbent assay (ELISA) to determine MMP-8 levels.^25^

In addition, the salivary samples of 12 periodontally healthy subjects were used as the reference for normal levels of MMP-8 (healthy group). MMP-8 levels were evaluated using Human MMP-8 ELISA kit (ZellBio GmbH, Germany) according to the manufacturer’s instructions based on the sandwich ELISA method using ng/mL. The ELISA plates were then assessed spectrophotometrically, and MMP-8 levels were calculated from the standard curves. The rates were measured in duplicate for each subject to improve accuracy and precision, the mean of which was used as the final data.

### Preparation of 20% propolis hydroalcoholic solution

Propolis extract was produced by a biotechnology company (Suren Tec., Tus, Mashhad, Iran). Propolis was frozen at -20°C and ground in a precooled mortar and pestle. The ground material was mixed with 99.8% (v/v) ethanol in a hermetically sealed glass container at a ratio of 1 g of propolis powder to 3 mL of ethanol. The containers were incubated in the dark for one week at room temperature, with constant stirring. The resulting ethanol solutions were centrifuged at 7000 g for 60 seconds, and the supernatants were then collected and filtered using the #4 Whatman paper filter. Ethanol-soluble components were collected by vacuum evaporation to dryness, and 20% (w/v) propolis hydroalcoholic solution was obtained by re-dissolving the extract in pure ethanol. The final solution was kept in hermetically sealed brown glass bottles at room temperature.^26^

### Statistical analysis

Thesample size was computed with Minitab software (Minitab Inc, State College, PA) by using a difference in the two-proportions test; considering the study results of Coutinho et al, α=0.5, β=0.2, and a minimum difference of 0.5, a sample size of at least 12 patients per group was determined.^23^ The data were analyzed using SPSS 22, Chi-squared test was used to compare gender distribution in groups. The Mann-Whitney test was used to assess and compare improvements in GI, PPD, AL, and BOP between the two groups after treatment. Finally, independent-sample t-test was used to compare ages, clinical parameters, and changes in MMP-8 levels between the two groups after treatment.

Wilcoxon signed-rank test was applied to assess the significance of changes in GI, PPD, AL, and BOP in each group postoperatively. One-way ANOVA was applied to assess the differences in MMP-8 levels between the three groups (two intervention groups and one control group). Post hoc Tukey tests were applied for pairwise comparisons of the groups. Type one error was considered at 5%, and P<0.05 was considered statistically significant.

## Results

**Figure 1:**
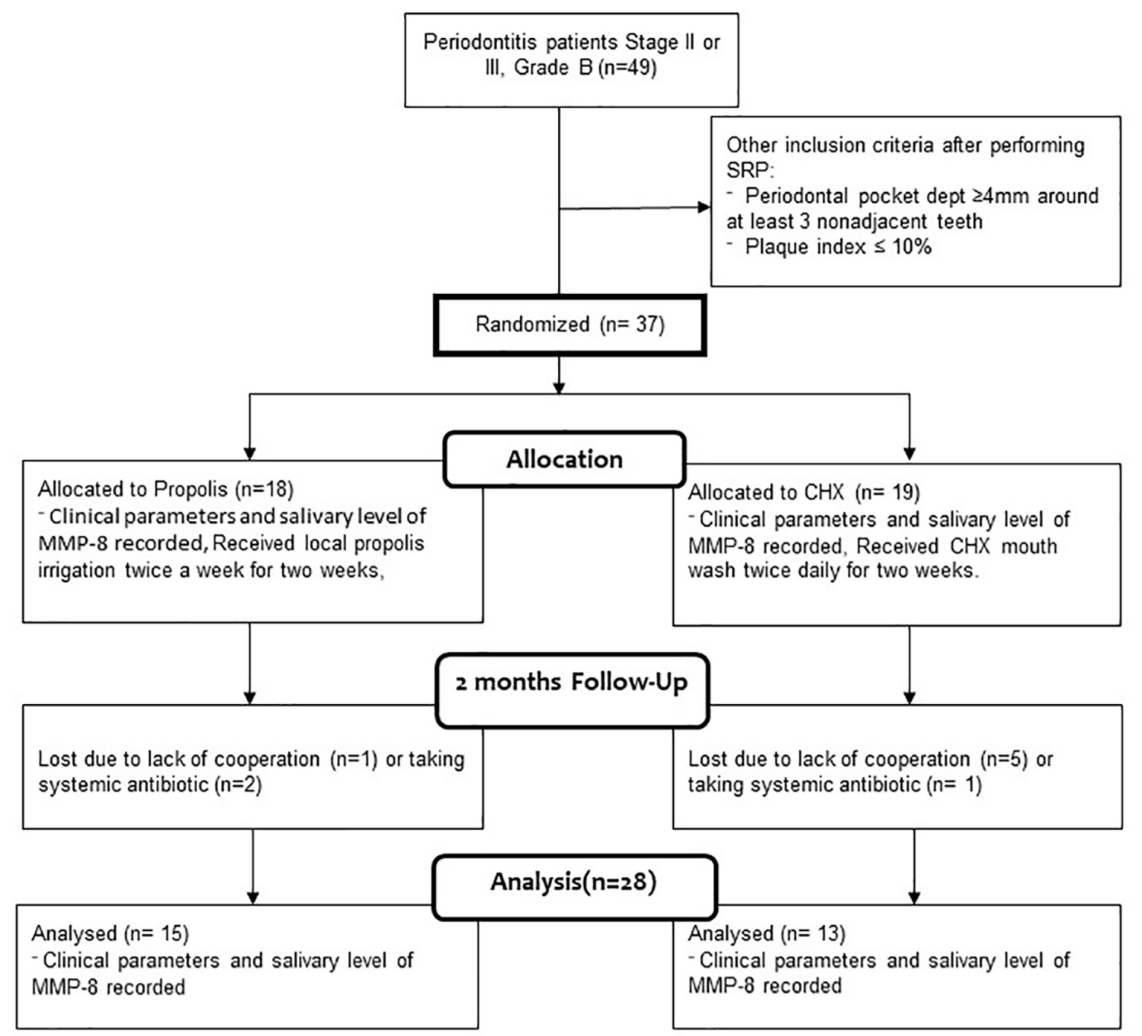
The Clinical trial’s flow diagram.

Thirty-seven patients met the inclusion criteria, but three were excluded during the study due to illnesses that needed systemic antibiotic therapy and six others due to lack of cooperation (Figure 1). Based on the new classification of periodontal diseases and conditions, patients were at stage II or III in terms of severity and grade B in terms of risk of progression.8 Based on the randomization of 28 patients with periodontitis (with a mean age of 46.39), 13 were assigned to the control and 15 to the study group; moreover, 12 healthy subjects were included to determine the normal salivary MMP-8 levels. Table 1 shows patients’ demographics. The three groups were almost matched regarding the number of males and females and their age (P>0.05).

**Table 1:** Demographic information of patients

**Groups**		**Propolis**	**CHX**	**Control**
**Gender**	Female	7	7	6
	Male	8	6	6
	Total	15	13	12
**Age (year)**	Min	25	25	25
	Max	67	61	60
	Mean±SD	46.9±15.3	47.0±12.7	45.1±11.3

CHX, chlorhexidine; SD, Standard deviation

After two months, improvements in GI were statistically significant in both groups (P<0.001), with no significant differences in this respect between the CHX and propolis groups (P=0.235, Table 2).

**Table 2:** Clinical periodontal parameters of propolis and CHX groups

**Clinical Parameters**	**Group**	**Baseline**	**2 months after operation**	**Changes Mean ± SD**	**P-Value**	
		**Mean±SD**	**Mean±SD**		**Within groups**	**Between groups**
BOP (Number of positive teeth)	Propolis	3.0±0.0	0.26±0.45	-	0.00	≥0.156
	CHX	3.00±0.0	0.61±0.65	-	0.00	
PPD (mm)	Propolis	5.06±0.44	3.33±0.56	1.73±0.22	0.00	<0.001
	CHX	4.69±0.43	3.61±0.56	1.07±0.22	0.00	
AL (mm)	Propolis	5.42±0.82	3.67±0.90	1.73±0.22	0.00	<0.001
	CHX	5.23±0.84	4.15±0.85	1.07±0.22	0.00	
GI (0-3)	Propolis	2.00±0.0	1.11±0.23	0.88±0.23	0.00	0.235
	CHX	2.00±0.0	1.21±0.22	0.78±0.22	0.00	
MMP-8 (ng/ml)	Propolis	274.30±69.44	174.95±76.84	99.35±82.08	0.00	0.63
	CHX	295.98±117.22	211.95±107.56	884.03±83.90	0.00	

SD, standard deviation; BOP, bleeding on probing; PPD, periodontal pocket depth; AL, attachment loss; GI, gingival index; MMP-8, matrix metalloproteinase-8

The decrease in PPD was statistically significant in both the CHX and propolis groups (P<0.001), with a significantly greater decrease in PPD in the propolis group than in the control group (P<0.001, Table 2). Table 2 shows that the postoperative decrease in AL was significant in both the control and propolis groups (P<0.001), and the mean decrease in AL in the propolis group was significantly greater than that in the CHX group (P<0.001). The improvement in BOP was significant in both the control and propolis groups (P<0.001). Although the frequency of points with negative BOP in the propolis group was higher, the difference between the propolis and control groups in this respect did not achieve statistical significance (P=0.156, Table 2).

**Figure 2:**
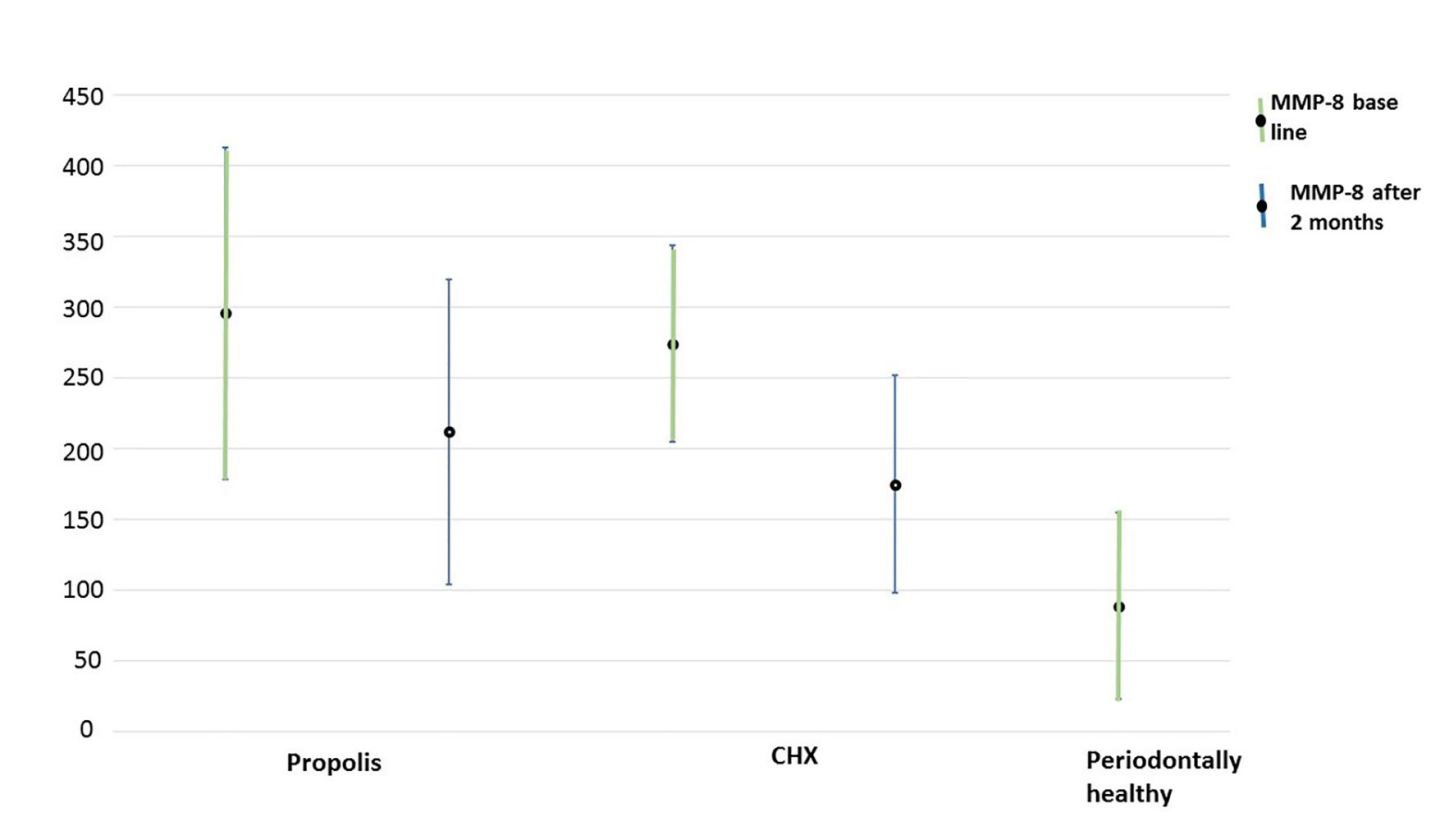
The mean (± standard deviation) of MMP-8 salivary levels (ng/mL) in the two groups of CHX and propolis at baseline and two months after treatment plus the mean (±standard deviation) MMP-8 salivary levels in the control group at baseline.

Table 2 displays the mean salivary MMP-8 levels in propolis and control groups. After treatment, salivary levels of MMP-8 significantly decreased in both groups (P<0.001). Although this decrease was higher in the propolis group, this difference between the two groups was not statistically significant (P=0.63). Furthermore, the mean of salivary levels of MMP-8 in the healthy group was significantly lower than those in the control and propolis groups (all periodontitis patients) at baseline (P<0.001, Figure 2).

## Discussion

The present study showed that the use of CHX and propolis as an adjunct to SRP significantly improved clinical parameters, and the salivary levels of MMP-8 decreased significantly in both groups after two months. Improvements in PPD and AL in the propolis group were significantly greater than those in the CHX group; however, there was no significant difference in GI, BOP, and changes in the MMP-8 levels between the CHX and propolis groups.

In the present study, the propolis administration intervals were in line with Coutinho et al,^23^ who prescribed subgingival propolis twice a week for two weeks, which resulted in a significant improvement in clinical parameters compared to the SRP treatment group. On the other hand, the US Food and Drug Administration recommends concentrations from 0.12% to 0.2% of CHX, 10‒15 mL for 30 seconds twice a day, for 15‒30 days.^27^ Therefore, in the current study, we aimed to compare a regular treatment (CHX) with subgingival propolis. In addition, we believe that prescription of propolis twice a week for two weeks can improve patients’ medication-taking compliance.

Propolis is a natural compound with several favorable biological properties.^14^ Sanghani et al, in a clinico-microbiological study, showed that subgingival irrigation with propolis hydroalcoholic extract in patients with chronic periodontitis significantly decreased the number of anaerobic bacteria and points with BOP.^18^ Furthermore, Nakao et al^19^ showed significant improvements in AL through the topical administration of propolis in a clinical trial. In the present study, propolis and CHX effectively reduced GI and BOP, with no significant difference in the mean changes between the two groups. Therefore, as concluded in previous studies, both treatments can be used to relieve the clinical manifestations of tissue inflammatory responses to periodontal disease.^28-30^ However, in terms of PPD and AL, while several studies have shown the beneficial effects of CHX,^31,32^ propolis was more effective. Therefore, it might be more promising in non-surgical periodontal treatment. In the current study, improvements in clinical parameters within the propolis group could be due to propolis activity against periodontal pathogens.^11,14,18,23,33^ In addition, the anti-inflammatory effects of propolis are effective to improve GI and BOP.^34^

Studies have shown that MMP-8 activity is a key sign of periodontitis. Significant associations have been reported between the activity of MMP-8 and AL, BOP, and PPD. Moreover, saliva is easily collected.^5,35^ Gupta et al^25^ found significantly higher levels of salivary MMP-8 in patients with periodontal disease relative to the control group. The same finding in our research showed that the salivary levels of MMP-8 were significantly higher in periodontal patients than in the periodontally healthy group.

In a study by Rai et al, high levels of salivary MMP-8 were significantly correlated to clinical parameters.^36^ In the present research, salivary levels of MMP-8 decreased significantly in both propolis and CHX groups two months after treatment. To the best of our knowledge, this is the first study showing the correlation between propolis administration in periodontitis patients and alterations in the salivary levels MMP-8. Thus, we propose that salivary MMP-8 levels can be used as an assessment tool that eliminates inter- and intra-clinician biases for future studies.

On the other hand, in the present research, the changes in the salivary levels of MMP-8 and BOP did not differ significantly between the CHX and propolis groups, consistent with Konopka et al^37^ study, showing no significant correlations between clinical parameters and the concentrations of humoral factors one month after treatment. However, longer follow-up of patients might have resulted in differences between the two groups in salivary levels of MMP-8 reduction.

In the current study, due to the evaluation of salivary levels of MMP-8, we were unable to assess the effects of propolis and CHX in the same patient, but we tried our best to standardize and balance the two groups and eliminate the confounders as much as possible.

## Conclusions

This randomized, single-blind study indicated that both CHX and propolis extract are successful adjuncts to SRP for treating deep periodontal pockets (≥4 mm) in periodontitis patients. However, improvements in clinical parameters were significantly greater in the propolis group. Propolis subgingival irrigation might thus be recommended for deep periodontal pockets in patients with periodontitis. Future studies with greater sample sizes and longer follow-ups are required to ensure the findings about the superiority of propolis to CHX for use in periodontitis patients as an adjunct to SRP.

## Authors’ contributions

REZ: conceptualization, methodology, investigation, writing the original draft, and supervision. MA: conceptualization, methodology, investigation, writing the original draft. HB: methodology, investigation, writing, reviewing, and editing. Iraj Mirzaii-Dizgah: methodology, investigation, writing, reviewing, and editing.

## Ethics approval

The study was approved by the Vice-Chancellor for Research, Tehran University of Medical Sciences (Ethical code: IR.TUMS.REC.1394.1066)

## Competing interests

The authors declare no conflicts of interest.
